# Well-hidden methanogenesis in deep, organic-rich sediments of Guaymas Basin

**DOI:** 10.1038/s41396-023-01485-y

**Published:** 2023-08-18

**Authors:** Diana P. Bojanova, Valerie Y. De Anda, Mojhgan A. Haghnegahdar, Andreas P. Teske, Jeanine L. Ash, Edward D. Young, Brett J. Baker, Douglas E. LaRowe, Jan P. Amend

**Affiliations:** 1 Department of Earth Sciences, University of Southern California, Los Angeles, CA USA; 2 Department of Marine Science, University of Texas at Austin, Austin, TX USA; 3 Department of Integrative Biology, University of Texas at Austin, Austin, TX USA; 4 Department of Geology, University of Maryland – College Park, College Park, MD USA; 5 Department of Earth, Marine, and Environmental Sciences, University of North Carolina, Chapel Hill, NC USA; 6 Earth, Environmental, and Planetary Sciences, Rice University, Houston, TX USA; 7 Earth, Planetary, and Space Sciences, University of California – Los Angeles, Los Angeles, CA USA; 8 Department of Biological Sciences, University of Southern California, Los Angeles, CA USA

**Keywords:** Biogeochemistry, Microbial ecology, Biogeochemistry, Soil microbiology, Metagenomics

## Abstract

Deep marine sediments (>1mbsf) harbor ~26% of microbial biomass and are the largest reservoir of methane on Earth. Yet, the deep subsurface biosphere and controls on its contribution to methane production remain underexplored. Here, we use a multidisciplinary approach to examine methanogenesis in sediments (down to 295 mbsf) from sites with varying degrees of thermal alteration (none, past, current) at Guaymas Basin (Gulf of California) for the first time. Traditional (^13^C/^12^C and D/H) and multiply substituted (^13^CH_3_D and ^12^CH_2_D_2_) methane isotope measurements reveal significant proportions of microbial methane at all sites, with the largest signal at the site with past alteration. With depth, relative microbial methane decreases at differing rates between sites. Gibbs energy calculations confirm methanogenesis is exergonic in Guaymas sediments, with methylotrophic pathways consistently yielding more energy than the canonical hydrogenotrophic and acetoclastic pathways. Yet, metagenomic sequencing and cultivation attempts indicate that methanogens are present in low abundance. We find only one methyl-coenzyme M (*mcrA*) sequence within the entire sequencing dataset. Also, we identify a wide diversity of methyltransferases (*mtaB, mttB*), but only a few sequences phylogenetically cluster with methylotrophic methanogens. Our results suggest that the microbial methane in the Guaymas subsurface was produced over geologic time by relatively small methanogen populations, which have been variably influenced by thermal sediment alteration. Higher resolution metagenomic sampling may clarify the modern methanogen community. This study highlights the importance of using a multidisciplinary approach to capture microbial influences in dynamic, deep subsurface settings like Guaymas Basin.

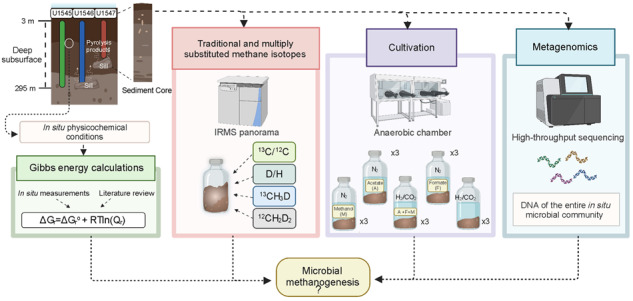

## Introduction

The deep marine subsurface biosphere is estimated to contain ~33–45% of the Earth’s microbial biomass, and 58% of this biomass is contained in sediments [1]. Various parameters have been used to define where the deep biosphere begins [2]; here we use the broad classification of any sediment deeper than 1 meter below the seafloor (mbsf). The microorganisms inhabiting deep marine sediments are major players in global element cycling [3], including the sequestration and mobilization of sedimentary carbon [4, 5]. The microbial production of methane (methanogenesis) alone accounts for the degradation of ~3–4% of the total organic carbon sinking to the seafloor [6]. Marine sediments are also the largest reservoir of methane on Earth [7] and the dominant supplier of the 5–25 Tg of CH_4_ (1–13% of natural emissions) released from the oceans every year [8]. Paleoclimatic records have linked past disturbances of this reservoir with abrupt climactic changes and consequent mass extinctions [9]. Yet, the distributions of the major methane sources (microbial, thermogenic, abiotic)  within marine sediments and what may influence source partitioning is just starting to be discovered [10, 11].

Despite methanogenesis being among the least exergonic catabolic strategies, it is nearly ubiquitous in marine sediments. Methanogens are typically outcompeted for substrates by sulfate reducers, and thus methanogenesis is not prominent until sulfate is depleted – denoting the sulfate methane transition zone (SMTZ) [12–14]. In the deep subsurface, however, methanogens and their contribution to methane production remain understudied. This is in-part because these sediments are hard to access and often poorly characterized, precluding both laboratory and theoretical studies of the in situ microbial communities.

**Fig. 1:**
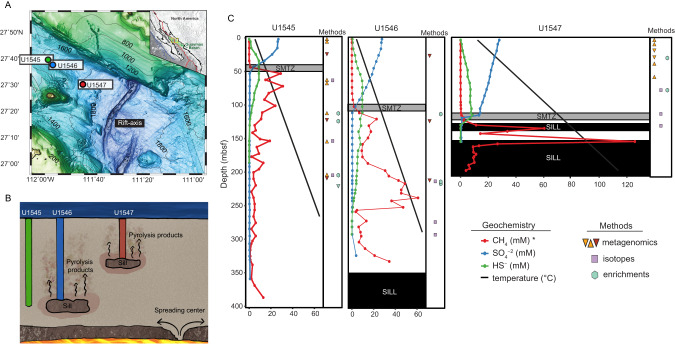
Background information on drill site geography, geochemistry, and sampling. **A** Bathymetry and geographical location of Guaymas Basin. The three sites of interest are indicated by circles: blue (U1545, reference site), green (U1546, site with deep, cooled sill), red (U1547, site with shallow, young sill). **B** Artistic interpretation of hydrothermal mobilization of buried organic carbon where magmatic sills intrude into the sediment. **C** Downcore geochemical profiles and depths from which samples for metagenomic, isotopic, and enrichment analyses were taken. The red, blue, and green dots and lines denote methane, sulfate and sulfide porewater concentrations, respectively, and the black lines depict temperature (Teske, Lizarralde, and Höfig 2021a; 2021b; 2021c). When triangles are: 1) upside down they indicate Sample set 1 of metagenomic samples (see methods for details) and 2) dark orange they indicate samples from hole C/D, rather than B (see methods for details). *Note that values of methane concentration at U1545 and U1546 are multiplied by 10. The shallowest methane peak at U1547 (~115 mbsf) is not easily seen because of the high concentration, deep peaks at this site.

In Autumn of 2019, IODP Expedition 385 drilled deep into the organic-rich sediments of Guaymas Basin (Gulf of California), providing access to pristine samples down to 540mbsf. Previous studies at Guaymas confirm high (mM) outgassing methane concentrations, microbially produced methane [15], and the presence of methanogenic lineages in surficial [16–20] and shallow sediments [21–23]. This prompted us to investigate methanogenesis in these deep subsurface sediments. The deep Guaymas subsurface is characterized by magmatic sill intrusions at various depths and locations which pyrolyze heavy-weight organics into lightweight and energy-rich substrates [24–26] that can be utilized by overlaying microbial communities [27, 28]. Thermal alteration also leads to large chemical and geothermal (up to 1000 °C/km) gradients [29], which create dynamic environments for overlaying microbial communities [28]. Such fluctuating environmental conditions are often associated with strong diversification [3, 30]. Thus, we focus on three IODP sites with varying degrees of sill-induced thermal sediment alteration (U1545=none, U1546=past, U1547=current) (Fig. 1A, B) to clarify the effects, if any, on the role of methanogens, relative to thermogenic and abiotic sources, in methane production.

Using shipboard measurements of porewater geochemistry and temperature, we identified distinct physicochemical patterns down-column at each site (Fig. 1C), including different thermal gradients and SMTZ depths, suggestive of variable microbial activity [12]. For a detailed description of the sites, see the supplementary text. We employ a multidisciplinary approach of thermodynamic calculations, metagenomic analysis, microbial enrichments, and methane isotope analyses, we address methanogenesis in the deep subsurface of Guaymas Basin for the first time. By including multiply substituted (^13^CH_3_D and ^12^CH_2_D_2_), in addition to traditional (^13^C/^12^C and D/H), methane isotope measurements we are able to resolve relative proportions of methane sources [31] and whether microbial methane was formed in an energy-rich (e.g., laboratory cultures) or energy poor environment (e.g., deep subsurface sediments) [32].

## Methods

### Sampling

Sediment samples were collected from Guaymas Basin, as part of the Integrated Ocean Discovery Program (IODP) Expedition 385 from three drill sites U1545, U1546, U1547 (Fig. 1B and Supplementary Table 1). As per IODP procedure, multiple holes (labeled A-D) were drilled at each site [33]. Samples were taken from holes B and C at U1545, which are 22 m apart, from holes B and D at site U1546, which are are 19 m apart and from hole B at U1547. The physicochemical patterns down-column are comparable between different holes of the same site [34, 35]. Samples of surface water and drilling fluid were collected as contaminant controls in DNA sequencing efforts. Cores for microbiological assays were either flushed with N_2_ and stored at 6 °C for future cultivation studies or immediately frozen at −80 °C for future genomics studies. Details on sample collection and processing are outlined in the IODP 385 Proceedings [36]. Measurements of in situ geochemistry, pressure, and temperature were conducted shipboard, as outlined in [29].

### Thermodynamic calculations

Gibbs energies (ΔG_r_) of the nine methanogenic catabolisms (reactions 1–9) listed below were calculated using  in situ physicochemical data (e.g., temperature, pressure, ionic strength, species activities) collected down the sediment column of each site:

1 $$4{{{{{{{\mathrm{H}}}}}}}}_{2({{{{{{{\mathrm{aq}}}}}}}})} + {{{{{{{\mathrm{CO}}}}}}}}_{2({{{{{{{\mathrm{aq}}}}}}}})} \to {{{{{{{\mathrm{CH}}}}}}}}_{4({{{{{{{\mathrm{aq}}}}}}}})} + 2{{{{{{{\mathrm{H}}}}}}}}_2{{{{{{{\mathrm{O}}}}}}}}$$

2 $${{{{{{{\mathrm{CH}}}}}}}}_3{{{{{{{\mathrm{COO}}}}}}}}^ - + {{{{{{{\mathrm{H}}}}}}}}^ + \to {{{{{{{\mathrm{CH}}}}}}}}_{4({{{{{{{\mathrm{aq}}}}}}}})} + {{{{{{{\mathrm{CO}}}}}}}}_{2({{{{{{{\mathrm{aq}}}}}}}})}$$

3 $$4{{{{{{{\mathrm{HCOO}}}}}}}}^ - + 4{{{{{{{\mathrm{H}}}}}}}}^ + \to {{{{{{{\mathrm{CH}}}}}}}}_{4({{{{{{{\mathrm{aq}}}}}}}})} + 3{{{{{{{\mathrm{CO}}}}}}}}_{2({{{{{{{\mathrm{aq}}}}}}}})} + {{{{{{{\mathrm{H}}}}}}}}_2{{{{{{{\mathrm{O}}}}}}}}$$

4 $${{{{{{{\mathrm{H}}}}}}}}_{2({{{{{{{\mathrm{aq}}}}}}}})} + {{{{{{{\mathrm{CH}}}}}}}}_3{{{{{{{\mathrm{OH}}}}}}}}_{({{{{{{{\mathrm{aq}}}}}}}})} \to {{{{{{{\mathrm{CH}}}}}}}}_{4({{{{{{{\mathrm{aq}}}}}}}})} + {{{{{{{\mathrm{H}}}}}}}}_{2({{{{{{{\mathrm{aq}}}}}}}})}$$

5 $$4{{{{{{{\mathrm{CH}}}}}}}}_{{{{{{{\mathrm{3}}}}}}}}{{{{{{{\mathrm{OH}}}}}}}}_{({{{{{{{\mathrm{aq}}}}}}}})} \to 3{{{{{{{\mathrm{CH}}}}}}}}_{4({{{{{{{\mathrm{aq}}}}}}}})} + {{{{{{{\mathrm{CO}}}}}}}}_{2({{{{{{{\mathrm{aq}}}}}}}})} + 2{{{{{{{\mathrm{H}}}}}}}}_2{{{{{{{\mathrm{O}}}}}}}}$$

6 $$4\left( {{{{{{{{\mathrm{CH}}}}}}}}_3} \right){{{{{{{\mathrm{NH}}}}}}}}_{2({{{{{{{\mathrm{aq}}}}}}}})} + 4{{{{{{{\mathrm{H}}}}}}}}^ + + 2{{{{{{{\mathrm{H}}}}}}}}_2{{{{{{{\mathrm{O}}}}}}}}_{({{{{{{{\mathrm{l}}}}}}}})} \to 3{{{{{{{\mathrm{CH}}}}}}}}_{4({{{{{{{\mathrm{aq}}}}}}}})} + {{{{{{{\mathrm{CO}}}}}}}}_{2({{{{{{{\mathrm{aq}}}}}}}})} + 4{{{{{{{\mathrm{NH}}}}}}}}_4^ +$$

7 $$2\left( {{{{{{{{\mathrm{CH}}}}}}}}_3} \right)_2{{{{{{{\mathrm{NH}}}}}}}}_{2({{{{{{{\mathrm{aq}}}}}}}})} + 2{{{{{{{\mathrm{H}}}}}}}}^ + + 2{{{{{{{\mathrm{H}}}}}}}}_2{{{{{{{\mathrm{O}}}}}}}}_{({{{{{{{\mathrm{l}}}}}}}})} \to 3{{{{{{{\mathrm{CH}}}}}}}}_{4({{{{{{{\mathrm{aq}}}}}}}})} + {{{{{{{\mathrm{CO}}}}}}}}_{2({{{{{{{\mathrm{aq}}}}}}}})} + 2{{{{{{{\mathrm{NH}}}}}}}}_4^ +$$

8 $$4\left( {{{{{{{{\mathrm{CH}}}}}}}}_3} \right)_3{{{{{{{\mathrm{NH}}}}}}}}_{2({{{{{{{\mathrm{aq}}}}}}}})} + 4{{{{{{{\mathrm{H}}}}}}}}^ + _{({{{{{{{\mathrm{aq}}}}}}}})} + 6{{{{{{{\mathrm{H}}}}}}}}_2{{{{{{{\mathrm{O}}}}}}}}_{({{{{{{{\mathrm{l}}}}}}}})} \to 9{{{{{{{\mathrm{CH}}}}}}}}_{4({{{{{{{\mathrm{aq}}}}}}}})} + 3{{{{{{{\mathrm{CO}}}}}}}}_{2({{{{{{{\mathrm{aq}}}}}}}})} + 4{{{{{{{{{{\mathrm{NH}}}}}}}}_{4}}^{+}}_{({{{{{{{\mathrm{aq}}}}}}}})}}$$

9 $$2\left( {{{{{{{{\mathrm{CH}}}}}}}}_3} \right)_2{{{{{{{\mathrm{S}}}}}}}}_{({{{{{{{\mathrm{aq}}}}}}}})} + 2{{{{{{{\mathrm{H}}}}}}}}_2{{{{{{{\mathrm{O}}}}}}}} \to 3{{{{{{{\mathrm{CH}}}}}}}}_{4({{{{{{{\mathrm{aq}}}}}}}})} + {{{{{{{\mathrm{CO}}}}}}}}_{2({{{{{{{\mathrm{aq}}}}}}}})} + 2{{{{{{{\mathrm{HS}}}}}}}}^ - _{({{{{{{{\mathrm{aq}}}}}}}})} + 2{{{{{{{\mathrm{H}}}}}}}}^ + _{({{{{{{{\mathrm{aq}}}}}}}})}$$

Values of the overall Gibbs energy of each catabolism was calculated as described elsewhere [37] and all species were assumed to be aqueous (aq). Apart from CO_2_, the in situ concentrations of the carbon substrates (acetate, formate, methanol, mono- di- tri-methylamine, and dimethylsulfide) were not directly measured. Instead, we calculated a range of ΔG_r_ for each catabolism using limit of detection concentrations as minimums and values from published studies on marine sediments as maximums [38–41], as shown in Supplementary Table 2. Activities were calculated by multiplying concentrations of the reactants and products by activity coefficients given in [42]. Since we assess several disproportionation reactions (e.g. reactions 2, 3, 5, 6, 7, 8, 9), values of ∆*G*_*r*_ are reported in units of kJ/mol-carbon-transferred, or kJ/mol C, to allow for standardized energetic comparisons.

### Metagenomics

#### DNA extraction and metagenomic sequencing

To prepare core samples for DNA extraction, they were thawed and the potentially contaminated outer 1 cm layer was removed in the flow hood under sterile conditions. The uncontaminated sediments were refrozen at −80 °C until extraction. DNA was extracted from 21 sediment cores, drilling fluid, and surface water sample using the FastDNA Spin Kit for Soil (MP Biomedicals, USA) according to the manufacturer’s instructions with the following adjustments: (step 5) debris was pelted in the centrifuge for 15 min at 14,000 × *g*; (step 15) Binding Matrix was resuspended in 30 µL DES for samples extracted in triplicate and 50 µL DES for samples extracted in 10 and 20-replicates. The eluent of samples extracted in 10 and 20-replicates were further concentrated using an Amicon Ultra-0.5 mL Centrifugal Filter. Resulting DNA was measured on a Qubit 2.0, using the Qubit dsDNA HD Assay Kit. Due to low biomass in the subsurface, DNA extractions were done either in triplicate, 10-replicates, or 20-replicates (see Supplementary Table 1).

Library preparation was done using a Nextera XT DNA Library Prep Kit (Illumina, San Diego, CA, USA) at the University of Delaware Sequencing and Genotyping Center. Paired-end sequencing of sample Set 2 (see Supplementary Table 1 for Sample Set members) was performed on-site on a NextSeq SBS platform (Illumina, San Diego, CA, USA) with fragments of 150 bp. Sequencing of sample Set 1 was carried out with insert sizes of 400–500 bp on a NovaSeq S4 (Illumina, San Diego, CA, USA) at the Davis Sequencing Center (University of California), resulting in 150 bp-long-paired-end reads. Sequences were demultiplexed and quality filtered for adaptor removal on site (see Supplementary Table 5 for sequencing results).

#### Metagenomic analysis

Sequences of sample Set 1 were trimmed with Trimmomatic v.0.35.6; [43]) as follows: trim adapters (mix of TruSeq3-PE-2 and Nextera) crop the first 14 bases, remove sequences shorter than 75 bp and set sliding window for quality less than 2. PCR replicates were removed using default settings of hts_SuperDeduper [44]. Sequences were assembled through MEGAHIT v1.2.9 [45] using the ‘meta-large’, k-min 27, k-max 127 options.

For sample Set 2, reads were trimmed for quality and adapters using Trimmomatic v0.39 [43] (parameters: leading:5; trailing,5; slidingwindow,5:15; removing sequences shorter than 50 bp, and cropping the 14 bases from the beginning of the read). TruSeq adapters were removed as follows TruSeq2-PE.fa:2:30:10:8:True. The quality of the reads was verified via FastQC v0.11.9 [46]. Interleaved reads were used as input for the assembly via MEGAHIT v1.2.9 [45] (parameters: –k-list 21,33,55,77,99,121 –min-count 2).

After quality filtering, both metagenomic sets were analyzed together. Short assembled contigs (<1000 bp) were removed. Assembly metrics were determined via Quast v5.0.2 11(53) (Supplementary Table 6). Postprocessing of metagenomic assemblies is outlined in the supplementary text.

#### Methanogenesis marker genes

Several approaches were used to detect methanogenesis marker genes in the metagenomic assemblies. First, the *mcrA* gene, encoding the alpha subunit of the methyl-coenzyme M reductase, was searched using HMM KofamScan v1.3.0 [47] MEBS v1.2 [48] (targeting K00399 and PF02745, respectively), graftM v0.13.1 [49], and mmseq2 v13.45111 [50] (against a custom *mcrA* database). The custom *mcrA* database comprises of 1216 sequences obtained from [51–53] and sequences from the MAGnify database (using search tools against COG4058 with the following parameters -E 1 –domE 1 –incE 0.01 –incdomE 0.03 –mx BLOSUM62 –pextend 0.4 –popen 0.02 –seqdb full). Second, we searched for trimethylamine and methanol methyltransferase genes (*mttB* and *mtaB* and respectively) as a proxy of methylotrophic methanogenesis. As references 823 *mttB* sequences were obtained from Uniprot [54] along with 12 previously describe *mttB* sequences from Brockarchaeota genomes (Supplementary Table 9) and 168 *mtaB* as described elsewhere [55].

The presence of genes encoding for methanol methyltransferase *mtaB* (PF12176) and trimethylamine methyltransferase *mttB* (PF06253) in the assemblies was annotated via MEBS v1.2. Only sequences with a percent identity >50% and >200 amino acids in length were kept for analysis. They were aligned with MAFFT v7.450 (default parameters) and refined with MUSCLE v3.8.425 (default parameters), then masked (50% gaps) in Geneious Prime 2020.0.5. The trees were generated using a using IQ-TREE v6 with the ultrafast bootstrapping option -bb 1000 and models LG+F+I+I+R6, LG+F+R10 and LG+F+I+I+R5s for *mcrA*, *mttB* and *mtaB*, respectively. Finally, sequences belonging to 38 arCOG methanogenesis marker genes [56] were obtained from the EggNOG database v6 [57] and identified their presence in the assemblies via DIAMOND v0.9.24.125 [58].

### Enrichments

Methanogenic cultivation experiments (150 total) were maintained over the course of two years. The inoculum was sediment slurry (6 cm^3^ of sample sediment to 40 mL of anaerobic methanogenic media [59] from sediment samples from in situ temperatures of roughly 20 and 50 °C at each of our study sites (see Fig. 1B). The slurry was then aliquoted in a volume ratio of 5% into media, in triplicate, that contained either 10 mM acetate, formate, methanol, 2 bar of 80:20 H_2_/CO_2_, or a mix of all four as substrate and was incubated at near in situ temperature. Enrichments were checked for cell growth via microscopy and methane production via gas chromatography over an incubation period of 2+ years (see Supplementary Table 3 for details).

We set up additional cultivations amended with either methanol, trimethylamine, DMS, or all three with a headspace of H_2_/CO_2_ [60] using sample U1546B_13H_2 (112 mbsf) at an in situ temperature of 30 °C to test for methylotrophic methanogenic pathways (in progress).

### Isotopes

Multiple isotopologue measurements of methane from freshly preserved sediment samples U1545B_8H_3, U1546B_29F_2, U1546B_49F_3, U1546B_54F_2, U1547B_9H_2, U1547B_21F_2, and U1547B_24F_2 were carried out at the University of Maryland Panorama Laboratory. Samples were purified as outlined in the supplementary text and then analyzed using the Panorama high-resolution mass spectrometer (Nu Instrument) at The University of Maryland – College Park. Ion currents of ^12^CH_4_^+^, ^13^CH_4_^+^, ^12^CH_3_D^+^, ^13^CH_3_D^+^, and ^12^CH_2_D_2_^+^ of methane gas samples were measured at mass resolving powers (MRP) of a minimum of 36,000 for clean separation of ^12^CH_2_D_2_ from ^13^CH_3_D, corresponding to an approximate entrance slit width of 35 μm. Isotopologue ratios were obtained using two different magnet current settings; one for obtaining δ^13^C and Δ^13^CH_3_D, and the other for δD and Δ^12^CH_2_D_2_ [61].

Multiple isotopologue measurements for samples U1545B_31F_2 and U1545B_19F_2 were carried out at the University of California - Los Angeles Panorama Laboratory. These are void gases sampled from freshly retrieved cores as described elsewhere [62]. Methane samples were purified on a vacuum line with an in-line gas chromatograph [63] and transferred via glass vial to the Nu Instruments Panorama for analysis. To fully distinguish ions of ^13^CH_3_D from ^12^CH_2_D_2_ from each other and their respective interferences, the Panorama was operated at a mass resolving power ≥40,000. Sample and standard bellows were adjusted to match ion current intensities (ranging from ~2–3 × 10^−10^ amps) in two stages. One centers on ^13^CH_3_D^+^ to simultaneously measure values of ^13^CH_4_^+^/^12^CH_4_^+^ and ^13^CH_3_D^+^/^12^CH_4_^+^ for up to 20 blocks of sample vs. standard integrations. The second centers on ^12^CH_2_D_2_^+^ to simultaneously determine values of ^12^CH_3_D^+^/^12^CH_4_ and ^12^CH_2_D_2_^+^/^12^CH_4_ for up to 40 blocks of sample vs. standard integrations.

## Results

### Energetics

**Fig. 2:**
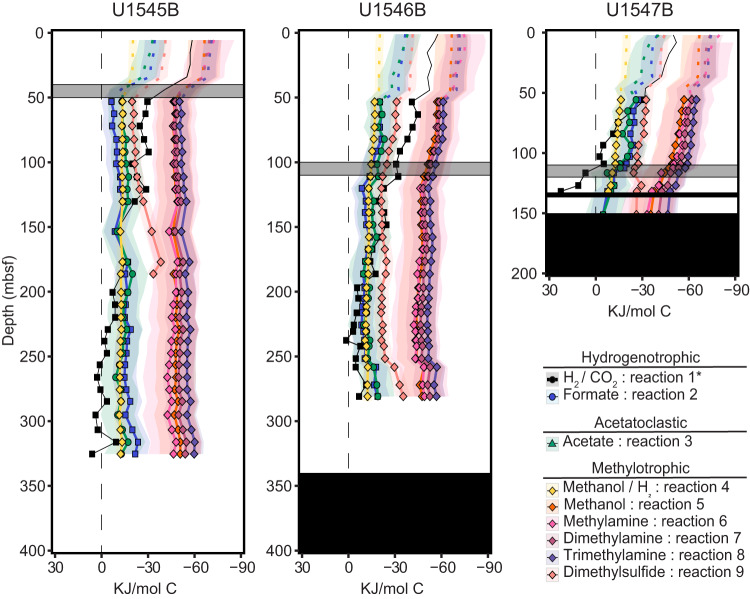
Gibbs energies, ΔG_r_, of the nine methanogenic catabolic pathways considered in this study as a function of sediment depth at the three study sites. The solid lines represent average ΔG_r_ values and the shaded envelopes around them refer to the possible range of ΔG_r_ for that reaction based on a range of substrate concentrations (see Supplementary Table 2). The dashed lines above 50 mbsf indicate methane concentrations were below the detection limit and an activity of 10^−7^ was used in the calculations. The vertical dashed black lines refer to ΔG_r_ = 0. The black blocks indicate a magmatic sill, and the grey blocks represent the sulfate-methane transition zone, SMTZ, at each site. *No ranges for the Gibbs energies of hydrogenotrophic methanogenesis are shown because in situ measurements of CO_2_ and H_2_ were used rather than a range of substrate concentrations (see methods).

The Gibbs energies of Reactions 1–9 were determined as a function of depth for the three sites (Fig. 2 and Supplementary Table 2). All are exergonic throughout the sediment column, except Reaction 1 which becomes endergonic below the SMTZ of U1547 (~100 mbsf) and around 250 mbsf at U1545 and U1546. At all sites, 4 of the 6 methylotrophic reactions (Reactions 5, 6, 7, 8) are the most exergonic, with average Δ*G*_*r*_ ranging between approximately −42 and −65 kJ/mol C. The reduction of methanol with H_2_ (Reaction 4), the disproportionation of DMS (Reaction 9), hydrogenotrophic (Reactions 1,2), and acetoclastic methanogenesis (Reaction 3) yield significantly less Gibbs energy (0 to −40 kJ/mol C.

### Metagenomics

**Fig. 3:**
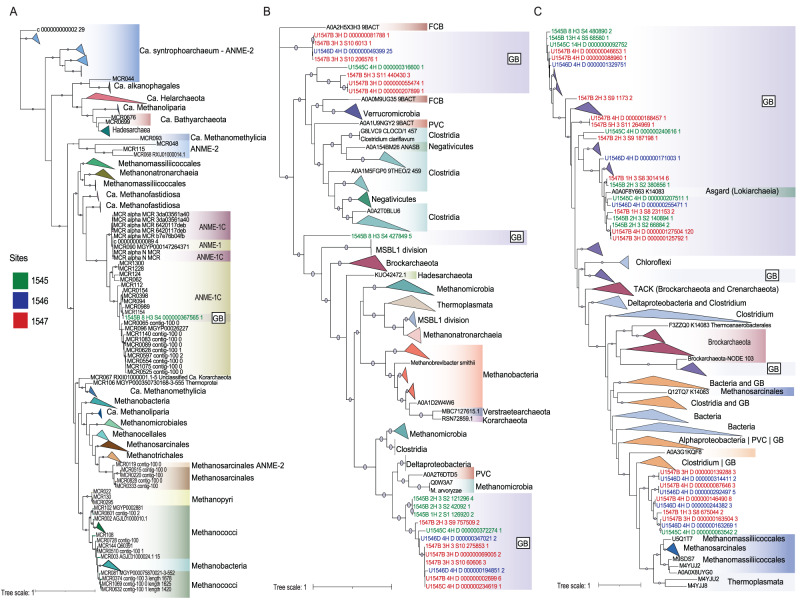
Phylogenetic evidence of methanogens in Guaymas subsurface. **A** A phylogenetic tree of the single methyl coenzymeM subunit A gene (*mcrA*) sequence identified in at 68.2 mbsf at U1545 along with 1216 McrA homologs as references (**B**) A phylogenetic tree of 21 methanol methyltransferase gene (*mtaB*) sequences identified in deep Guaymas sediments, along with 168 *mtaB* homologs as references. Those phylogenetically related to known methanogens (12 total sequences) are highlighted in red. **C** A collapsed phylogenetic tree of 1079 trimethylamine methyltransferase genes (*mttB*) identified in deep Guaymas sediments and 823 reference *mttB* homologs. Those that are monophyletic to known methanogens (10 sequences) are highlighted in red. Text colored in green, blue, red indicates sequences were recovered from sites U1545, U1546, U1547, respectively. See Supplementary Table 1 to discern the depth and in situ depth of the sample from which each sequence homolog originates. The phylogenies were constructed using IQ-TREE V6. Models LG+F+I+I+R6, LG+F+R10 and LG+F+I+I+R5s were selected for *mcrA*, *mttB* and *mtaB*, respectively, using the Bayesian Information Criterion (BIC). Bootstrap values were calculated using non-parametric bootstrapping with 1000 replicates represented by purple circles, where only bootstraps >90 are shown.

Using several methods on our 21 metagenomic assemblies, we identified one *mcrA* at site below the SMTZ of U1545 (68.2 mbsf) (see Supplementary Table 7). This *mcrA* is monophyletic to known methane oxidizing *Methanophagales* (previously ANME-1) lineages (Fig. 3A). The *mcrA* did not assemble into any metagenome-assembled genomes (MAGs), so they are not discussed in this study and will instead be reported on in upcoming publications from IODP 385. Furthermore, none of the sediment assemblies contained more than 19 of the 38 methanogenesis marker genes that were surveyed (Supplementary Table 8).

**Fig. 4:**
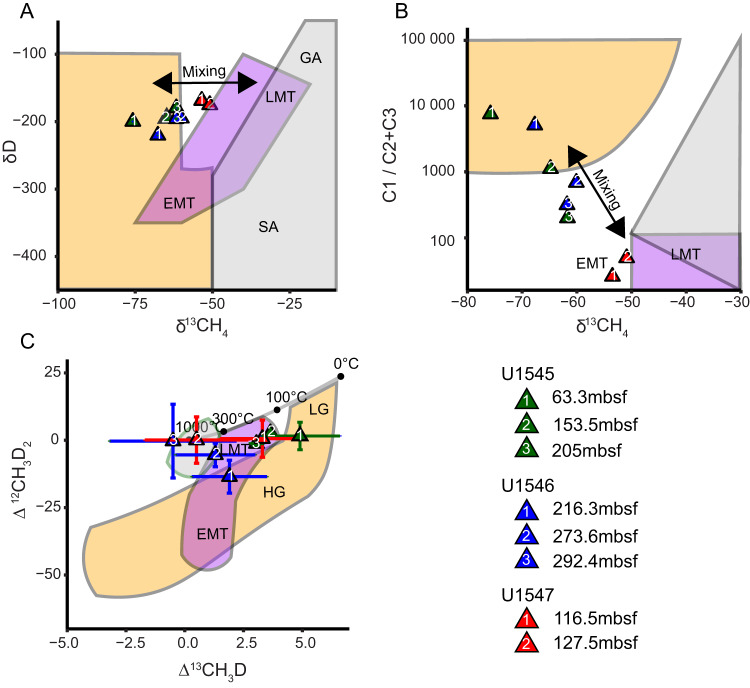
Isotopic and light hydrocarbon measurements of methane samples. **A** Traditional (δ^13^C vs. δD), (**B**) multiply substituted (^13^CH_3_D vs. ^12^CH_2_D_2_) isotopic measurements and associated errors, **C** Bernard plot (δ^13^C vs. C1/(C2 + C3) of methane samples taken from the depths indicated in Fig. 2. The orange, purple and grey polygons in each panel refer to regions in isotopic and chemical space that are indicative of biotic, thermogenic (LMT late maturity thermogenic, EMT early maturity thermogenic), or abiotic sources (SA serpentinization abiotic, GA geothermal abiotic) of methane, respectively (see text for citations). The solid grey line in (**C**) represents the equilibrium distribution of the indicated methane isotopologues from 0–1000 °C. Measurement error is indicated by the error bars around each point. See the legend for the meaning of samples shapes, colors and numbers.

We found multiple sequence homologous to *mtaB* and *mttB* within the assemblies. Phylogenetic analysis indicated some of these methyltransferase genes are related to known methanogens (Fig. 4B, C). Of the 21 *mtaB* sequences, 12 form a distinct and novel clade that is affiliated with *Methanocella arvoryzae*. These sequences span multiple depths above the SMTZ of all three sites. One sequence was found in the same sample as the identified *mcrA* sequence (U1545 at 68.2 mbsf) and phylogenetically clustered with the *MSBL-1 division*, which have been proposed to carry out methanogenesis [64]. Despite the wide diversity of trimethylamine methyltransferase genes (*mttB*) identified (1079 total), only 10 are monophyletic to known methanogenic *Methanomassiliicoccales*.

### Enrichments

150 microbial cultivation experiments were carried out at 30 and 50 °C (in situ temperatures) for >2 years (Supplementary Table 3). Substrates in the growth media were acetate, formate, methanol, H_2_/CO_2_, or a mixture of all four, with sediment slurries from various depths (Fig. 1B) serving as inoculum. Neither cell presence/growth nor methane production were detected in any of the enrichments throughout the multi-year period.

### Methane isotopes

Results and precision from mass spectrometry measurements are reported, alongside light hydrocarbon ratios, in Supplementary Table 4 and plotted in Fig. 4A–C. Plotting is done with respect to microbial, thermogenic, and abiotic regions as identified from natural methane samples for δ^13^C vs. δD and δ^13^C vs. C1/(C2+C3) [65, 66] and from a combination of natural and laboratory methane samples for Δ^13^CH_3_D vs Δ^12^CH_2_D_2_ [61, 67, 68]. The isotopologue plots include the recently proposed disequilibrium thermogenic field [69] and equilibrium microbial fields (supporting both AOM and methanogenesis) [63, 70]. δD values increase with depth from −200 to −180‰ in U1545, from −220 to −196‰ in U1546, and from −169 to −175‰ in U1547. Similarly, δ^13^C also increases with depth from −76 to −62‰ in U1545, from −68 to −62‰ in U1546, and from −54 to −51‰ in U1547 (Fig. 4A). Δ^13^CH_3_D decreases with depth from 4.8 to 3.0 in U1545, from 1.9 to −0.5 in U1546, and from 3.3 to 0.5 in U1547. There is little change with depth in values of Δ^12^CH_2_D_2_ at U1545 (from 1.6 to −1.22) and U1547 (from 6.9 to 8.6), while at U1546 values increase (from 6.1 to 13.7) (Fig. 4B). The isotopologue result for the shallowest sample at U1547 (75.5 mbsf) plots outside the known clumped methane limits of both Δ^13^CH_3_D vs Δ^12^CH_2_D_2_, along with very large error bars for both isotopologues (see Supplementary Table 4), and thus will not be discussed in this study. The ratio of C_1_/C_2_+C_3_ decreases with depth at sites U1545 (from 7500 to 200) and U1546 (from 5083 to 316) but at U1547 does not trend with depth and remains consistently low (ranging from 26 to 57) (Fig. 4C).

## Discussion

### Microbial contribution to methane production

Unlike most sedimentary marine basins, Guaymas Basin is characterized by the emplacement of magmatic sills originating from a rift axis into thick, organic-rich sediments. This provides a unique opportunity to survey sediment columns with varying levels of thermal sediment alteration for patterns of methane source contributions. We focus on the relative contribution of microbial methane through measurements of methane isotopes (traditional and multiply substituted) and light hydrocarbon ratios as a function of depth and sill presence. Due to thermocatalytic cracking organic-rich sediments during sill emplacement, much, if not all, of the non-microbial methane at Guaymas is likely to be thermogenic in origin. However, methane may be abiotically sourced from the sill itself upon emplacement [71]. To not discount the latter option, we herein collectively refer to thermogenic and abiotic sources of methane as “non-microbial”. Furthermore, we only discuss the partitioning of methane sources below the SMTZ of each site, in what we will refer to as the methanogenic zone (MZ), as low methane concentrations did not permit isotopic measurements in shallower sediment.

The results of our methane isotope measurements suggest methane sources at all sites shift from microbial to non-microbial as a function of depth in the sediments. This interpretation is based on decreasing Δ^13^CH_3_D and (C1/C2+C3) and increasing δ^13^C values with depth (Fig. 4; Supplementary Table 4). These trends have been observed in previous sedimentary settings, where in shallower depths of the MZ methanogens carry out methanogenesis [56, 72, 73]. Then, at depths (0.7–5.0 kmbsf) where temperatures exceed 150 °C, non-microbial methane production becomes abundant [74]. At U1546 and U1547, a strong non-microbial signal is present at relatively shallow depths, likely derived from the sill emplacements. Although sill-induced sediment disturbance was not found at U1545, a thin, deep sill at 540 mbsf could be a source of the non-microbial methane. Alternatively, non-microbial methane could have diffused laterally from the deeply emplaced sill of the neighboring U1546.

**Fig. 5:**
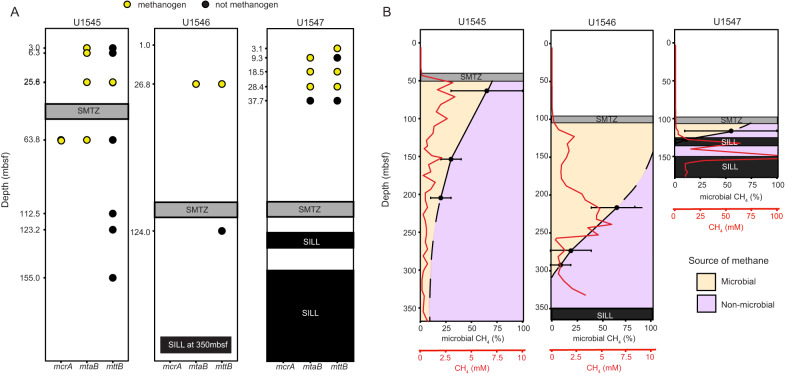
Presence of key methylotrophic methanogenesis genes and the percent microbial methane (PMM) throughout the sediment column of each site. **A** The presence of sequences related to the methylotrophic methanogenesis genes *mcrA*, *mtaB*, and *mttB* at the metagenomically surveyed depths of sites U1545, U1546, and U1547 is specified by circles. Black circles indicate if a given sequence is present and yellow circles indicate if the sequence is phylogenetically related to  known methanogen lineages. **B** The PMM as a function of depth based on mixing model results (see supplementary text for details). The average PMM of each measured sample is represented by black dots, with error bars indicating the full range of possible PMM. The dashed black line is an extrapolation of the patterns seen from the measured samples. The red line is the concentration of methane in mM as a function of depth.

To determine the percentage of microbial methane (PMM) in each sediment sample, we constructed isotopic mixing models (Supplementary Figs. 1–3) that take into consideration δ^13^C, δD, Δ^13^CH_3_D and Δ ^12^CH_2_D_2_ as previously described [67, 75]. The PMM results summarized in Fig. 5A are a combination of two mixing models: one with microbial and thermogenic endmembers, and another with microbial and abiotic endmembers, as described in the supplementary text. In the shallow sediments of the MZ of U1545, the average PMM is 75%. With depth, PMM and methane concentrations decreases in tandem, suggesting microbial processes are the predominant source of methane at this site.

Compared to U1545, U1546 has an SMTZ twice as deep and cell densities there are two orders of magnitude lower (10^6^ cell/cm^3^ vs. ~10^4^ cell/cm^3^) [76]. Yet, we find PMM is ~35% higher in the upper section of the MZ of U1546 than of U1545 and remains >50% down to the crest of the second methane peak (~225 mbsf). This implies a more prominent and active methanogenic community at U1546. Indeed, isotopologue values at U1546 are disequilibrated. Disequilibrated isotopologues are associated with energy-rich environments, where microbial methane is rapidly produced [31, 61, 68, 77] (see supplementary text for discussion); while the microbial endmembers of U1545 and U1547 are of near-equilibrium isotopologue values typical of the slow, energy-limited deep subsurface biosphere [63, 70, 78–80]. Because of the deep MZ at U1546, the methanogen community is close enough to the sill to capitalize on the buffet of energy-rich substrates diffusing upwards and may thus produce methane more rapidly. The uppermost methane peak at U1546 is half the concentration of the middle peak (~2.5 mM vs ~6 mM, respectively), yet the same amount of methane is microbially produced at both peaks (PPM of 100% vs 50%, respectively) despite variable cell densities [76]. In other words, the closer to the sill, the more productive methanogenesis is. The bioavailable substrates that were released when the sill first intruded have been proposed to no longer be available to the modern microbial community [81]. As such, much of the microbial methane at U1546 may have been produced by previous generations of methanogens.

By the top of the MZ at U1547, temperatures already exceed 70 °C, limiting the microbial community to thermophilic and hyperthermophilic members. Indeed, cell densities at this depth are only about 10^2^ cell/cm^3^ [76]. Yet, both the methane concentration (~2.5 mM) and PMM (55%) are comprable to those in the shallow MZ of U1545 and U1546. It should be noted that the PMM at U1547 has large error bars (45%), but even with the most conservative estimate, the microbial signal is at least a 10%. Methanogenic isolates from surficial hydrothermal sediments of Guaymas Basin can persist at temperatures 80–110 °C [28], but isotopic expression of methanogenesis has only been observed in cool sediments [18]. A recent radiotracer analysis of deep subseafloor methane from the Nankai Trough established high temperatures (>80 °C) may stimulate high cell-specific catabolic rates and biomass turnover in the methanogenic community [82]. We posit that while the number of methanogenic cells at U1547 may be low, their per-cell activity may be quite elevated. Within 20 m into the MZ, the microbial methane signal quickly depletes, likely due to strong inputs of sill-produced methane from below. Unlike at U1546, the proximity of the MZ to the sill at this site appears detrimental to the methanogen community. Equilibrated isotopologue values in the shallow part of the MZ corroborate this. Overall, microbial methane seems to be a significant proportion of the overall methane at all three sites, with evidence that the methanogen community at U1546 is (or was) the most active.

### Bioenergetics and methanogenic pathways

Although all nine of the methanogenesis reactions considered in this study could provide energy for microorgansims in Guaymas Basin sediments (Fig. 2), the likelihood of particular reactions being catalyzed in specific sediment sections are best understood by incorporating complementary information. For instance, it is known that sulfate reducers typically outcompete hydrogenotrophic and acetoclastic methanogens for H_2_ and acetate, and therefore, these varieties of methanogens are primarily active in sediments below the SMTZ [13, 83]. Above the SMTZ, however, methylotrophic methanogens are suggested to coexist with sulfate-reducing microbial communities, which do not compete for methylated C1 substrates [84–86]. As such, it is noteworthy that four of the six methylotrophic methanogensis reactions (Reactions 5–8) yielded more Gibbs energy than the canonical hydrogenotrophic and acetoclastic reactions (Reactions 1–3), hinting that methylotrophy may be the major methanogenic catabolism even below the SMTZ of deep Guaymas sediments. Nevertheless, as demonstrated by an isolate-based laboratory experiment, metabolically flexible microorganisms do not always preferentially utilize the growth substrate with the maximal energy yield [87].

A recent modeling study reported near-equilibrium isotopologue values for methanogenic reactions with low energy yields (<20 kJ/mol) and disequilibrium values for reactions with high energy yields (>20 kJ/mol) [70]. As noted above and in the supplementary text, microbial methane at U1545 and U1547 has isotopologue values near-equilibrium, while values out of equilibrium are observed at U1546. It would follow that microbial methane production at U1545 and U1547 would result from low-energy catabolisms (e.g., Reactions 1–4, 9), where Reactions 4 and 9 are methylotrophic pathways; while at U1546, methane is produced from the higher energy methylotrophic catabolic suite (Reactions 5–8). Thus, we further investigated methanogenesis, with a spotlight on methylotrophyic mathanogenesis, through metagenomic sequencing and cultivation attempts.

### Sequencing and cultivation of Guaymas Basin methanogens

Amongst our 21 sequencing assemblies, we only found one *mcrA* sequence at U1545 (68.2 mbsf), which we infer may have the potential to perform methanogenesis even below the SMTZ. The *mcrA* sequence phylogenetically clusters with the *Methanophagales*, which are known to perform methane oxidation but have also been proposed to be capable of methanogenesis based on geochemical conditions [88, 89]. A recent paired enrichment and transcriptomic study from White Oak River estuarine sediments indeed concluded that members of the *Methanophagales* perform methane oxidation within the SMTZ and methanogenesis below it [90]. We also found two scaffolds, each identified by BLAST as *Methanophagales* and *Vestraearchaeota*, from the same sample each contained a *mcrB* and a *mcrG* subunit (Supplementary Table 7). A recent sequencing study directly targeted *mcrA* and *Methanophagales*, rather than using metagenomics, in the same Guaymas Basin sites as this study but different depth horizons. They identified a few homologous sequences above the SMTZ, which consisted of a few methanogenic lineages and the *Methanophagales* [91]. As seen in Fig. 3A, the *Methanophagales* are sister to the *Methanofastidiosa*, *Methanomassiliicoccus*, and *Methanonatronarchaeia*, all of which are methylotrophic methanogenic lineages. However, experimental evidence of *Methanophagales* performing methylotrophic methanogenesis is currently lacking, and circumstantial bias may affect inferences about functionality from phylogenetics [92].

Methylotrophic methanogens have been detected in small proportions in surficial sediments of Guaymas Basin [20] and in other deep marine sediment environments [84–86]. We identified novel clades of 12 *mtaB* and 10 *mttB* sequence homologs in our assemblies that are monophyletic with known methylotrophic methanogens (Fig. 3B and C, respectively). These sequences mapped back to sediments located above the SMTZ of all three sites, in the sulfate-reducing zone (Fig. 5B). These findings add to the mounting evidence that methanogens can prosper in the sulfate-reducing and possible other, zones by utilizing non-competitive compunds [85, 93]. We also identified one *mtaB* sequence from the same sample where we found the *mcrA* homolog (below the SMTZ of U1545). This sequence phylogenetically clusters amongst the *MSBL-1 division*, which have been proposed to carry out methanogenesis [64]. Competition for methylated substrates may play a limiting role in the deep subsurface methanogen community at Guaymas. Most of the methyltransferase homologs (11 *mtaB* and 1069 *mttB*) do not cluster with methanogens, span the entire sediment columns, have a wide phylogenetic diversity, and form novel clades. Various homoacetogens have the capability to utilize methylated substrates, but direct competition with methanogens has yet to be shown [94]. Further investigation is needed to identify these lineages and to determine if they are actively competing with methanogens for methylated compounds.

Our metagenomic data suggest that methanogens account for a very small portion of the total microbial community in Guaymas Basin’s deep sediments. Previous amplicon and metagenomic studies of shallow Guaymas sediments [17] and organic-rich deep sediment sites, such as the Peru [95] and Cascadia Margins [96], also found little genomic support for methanogens despite geochemical and isotopic evidence of their presence. It was reasoned that this may be due, in part, to a distinct composition of lineages and metabolic capabilities in deep subsurface communities. In our study, we find novel clades of *mtaB* and *mttB* sequences that support this argument. A study focused on shallow sediments of Ringvent (where U1547 is located) also found a low representation (0.1–0.01%) of methanogens in their 16 S rRNA gene amplicon data. Low detection of methanogens in our deep subsurface study may be because our sampling resolution for metagenomics was low overall and variable between sites (Fig. 1c). At U1546, where most microbial methane appears to have accumulated, only two depths below the SMTZ were sampled. Due to the steep thermal gradient at U1547, cell densities dropped off more quickly and sequencing was only possible above the SMTZ. At all sites, the SMTZ is located at depths where cell densities are low and little DNA can be extracted, at least with current techniques. At the sill-influenced U1546 and U1547, the SMTZ is even deeper, further limiting accessibility to DNA from the methanogenic zone.

We also initiated 150 methanogenic batch enrichments but detected no cell growth or methane production even after 2+ years. To date, enrichments of deep marine subsurface methanogens have been limited [13, 97–99]. These results may be explained by the extremely low energy fluxes [100] used by subsurface microorganisms, their slow (sometimes non-existent) doubling times [101, 102] that do not translate well to laboratory settings, or they can be understood as evidence that methanogens are part of the rare biosphere in the Guaymas Basin subsurface.

## Concluding remarks

We combine geochemical data with thermodynamics, metagenomics, traditional and multiply substituted methane isotopes, and microbial culturing to evaluate methanogenesis in deep, subsurface sediments of Guaymas Basin (see Supplementary Fig. 4 for graphical concluding remarks). We focus on three drill sites (U1545, U1546, U1547) with variable thermal sediment alteration, which have not, to date, been microbiologically surveyed. Isotopic measurements reveal significant proportions of microbial methane below the SMTZ at all sites. However, this microbial signal is overprinted by non-microbial methane at variable rates with depth, depending on the presence/absence and age/depth of a sill emplacement at each site. We observe the most microbial methane at U1546, where a deep sill (~350 mbsf) has thermally re-equilibrated with the surrounding sediments. The combination of moderate temperatures and an initial influx of bioavailable pyrolysis products may have selected for specialists that can accommodate the new physicochemical conditions and produce methane more rapidly at U1546 [103]. Multiply substituted methane isotope measurements indeed suggest the microbial methane at U1546 was produced in a more energy-rich environment and through pathways with higher Gibbs energy yields than at U1545 or U1547. While isotopic evidence of microbial methane remains hidden above the SMTZ, due to low methane concentrations, Gibbs energy calculations reveal various methanogenic reactions are exergonic throughout the entire sediment columns of the three sites, with methylotrophic methanogenesis pathways yielding the most energy. The identification of *mtaB* and *mttB* sequences that are phylogenetically related to methanogens also supports the potential dominance of methylotrophic type methanogenesis, particularly above the SMTZ; suggesting methanogens may be even more widespread in marine sediments than traditionally thought. Overall, however, metagenomic analysis and cultivation attempts yield little evidence of methanogens, suggesting they do not represent a major proportion of the overall microbial community at Guaymas. Better DNA extraction techniques and higher sampling resolution may reveal more about this deep and rare biosphere. Furthermore, genomic and cultivation-based evidence of methanogens in subsurface Guaymas may remain hidden because these approaches only capture the currently active microbial community, while thermodynamics and isotopic measurements inform on methane accumulation throughout geologic time. Through our multidisciplinary approach we conclude low abundance methanogenic communities have been active in deep sediments over geologic time, with variable influences from thermal sediment alteration, leading to the accumulation of the observed microbial methane in the deep subsurface sediments of Guaymas Basin. Methanogens maintain their crucial ecological role, even in dynamic sediment environments such as Guaymas Basin, where they are presented with obstacles such as thermal stressors, physical displacement, and potential substrate competition.

### Supplementary information

Supplementary Tables

Supplementary Text and Figures

## Data Availability

The metagenomes generated during and analysed during the current study are available in the National Center for Biotechnology Information (NCBI) Genbank database under BioProject PRJNA909197 with accession numbers SRR22580929-SRR22580947 and SRR25383461- SRR25383464. As mentioned in the results, while metagenome assembled genomes (MAGs) were generated and searched for methanogens, they are not discussed in this study and will instead be reported on in upcoming publications from IODP 385. As such, these MAGs are not yet publicly available.
