# Supplementary material for: Well-hidden methanogenesis in deep, organic-rich sediments of Guaymas Basin
Source: ISME J. 2023 Aug 18;17(11):1828–38. doi: 10.1038/s41396-023-01485-y (PMC10579335; doi:10.1038/s41396-023-01485-y)
Supplement: Supplementary file 2 — Supplementary Text and Figures [file 41396_2023_1485_MOESM2_ESM.pdf]

SITE INFORMATION

Guaymas Basin, Gulf of California, is a young, active rift basin with a dynamic sedimentary environment that is influenced by magmatic sill intrusions (Figure 1). Unlike mid-ocean ridges, where little sediment accumulates, Guaymas Basin is blanketed by hundreds of meters of organic-rich (2-4%) sediment due to productive overlying waters (1,2). This thick sediment cover prevents rift magma from extruding onto the seafloor, as is common at mid-ocean ridges. Instead, the magma intrudes into the sediment, forming lateral magmatic sills (3). The intrusions pyrolyze and remobilize deeply buried organic carbon and replenish the sediment column with various dissolved substrates (e.g., methane, carbon dioxide, low-molecular weight organic acids, ammonia) (4–6). These can then be utilized by microorganisms that were limited to ancient, low-reactivity carbon sources before the thermal transformation of sediments (7–9). The thermally altered sediments are characterized by large chemical and geothermal (up to 1000°C/km) gradients (10) which, in combination with advective and diffusive gas and fluid flow towards the surface, create dynamic environments for local microbial communities (9).

While previous drilling during DSDP Leg 64 focused on the axial regions of Guaymas Basin, IODP Expedition 385 explored off-axis regions, following the discovery of active magmatic emplacement >50km from the rift axis (11). The focus of this study is on three off-axis sites (U1545, U1546, U1547) in the NW trough of Guaymas Basin with distinct thermal histories (Figure 1b). U1545 and U1546 are ~1km apart and located ~52km northwest of the spreading center (12,13). Despite their proximity and virtually identical sedimentation histories, they have different levels of sediment alteration. The sediment succession at U1545 appears undisturbed despite evidence of a thin sill at ~540mbsf, making it ideal as a thermally unaltered reference site. At U1546, on the other hand, sedimentary disturbance is attributed to the thick, albeit now cooled,

sill at ~350mbsf. The third site, U1547, is located ~20km NW of the spreading center and is experiencing active thermogenic alteration via a shallow (~75mbsf), young sill. U1547 is located inside of the circular hydrothermal mount known as Ringvent (14,15). While located in a different location than U1546, U1547 may be indicative of conditions at U1546 when its sill was first emplaced.

Using shipboard measurements of porewater geochemistry and temperature from hole B, we identified distinct physicochemical patterns down-column at each site (Figure 2), including different temperature gradients and SMTZ depths, suggestive of variable microbial activity (16). The temperature gradient at U1547 (511°C/km) is nearly double that at U1545 and U1546 (221 and 225°C/km, respectively). The similar gradients at U1545 and U1546 implies the sill at U1546 had reached thermal equilibrium with the surrounding sediment (14). All three temperature gradients are significantly higher than the global average (25°C/km). The SMTZs are at ~45 mbsf at U1545 and at ~110mbsf at U1546 and U1547 (both of which have magmatic sills present). At all three sites, the methane levels below the SMTZ are as high as 2.5-3mM, but at U1546 and U1547 two additional peaks are present at greater depths. At U1546, methane concentrations reach 6.09 mM at 237.6 mbsf and 3.4 mM at ~332.3 mbsf. The methane levels at U1547 are 1-2 orders of magnitude higher, reaching 60.4 mM at 131.9 mbsf and 125.7 mM at 151.2 mbsf. We build this study on these preliminary findings.

METHODS

Metagenomic binning

The scaffolds from the MEGAHIT assemblies (>2000 bp) were binned using MetaBAT v2.12.1 (Kang et al., 2015) and CONCOCT v1.1.0 (Alneberg et al., 2014), and resulting bins were assessed

and filtered using DAS Tool v1.1.2 (Sieber et al., 2017). The accuracy of all the bins was evaluated by calculating the percentage of completeness and gene duplication using CheckM lineage_wf v1.0.5 (Parks et al., 2015). Bins which were >50% complete but > 10% redundant were manually refined using Anvi'o's anvi-refine function. Ultimately, only bins >50% complete and <10% redundant were kept in the dataset. Bins assembled from scaffolds which map to the drilling fluid and blank samples were used to decontaminate the overall bin dataset. The finalized bins were surveyed for mcrA sequences found in the general assembly that may have mapped back to them. We did not find this to be the case. As such, the bins are not discussed or reported in this study.

Isotopic Analysis

Freshly preserved sediment samples U1545B_8H_3, U1546B_29F_2, U1546B_49F_3, U1546B_54F_2, U1547B_9H_2, U1547B_21F_2, and U1547B_24F_2 were prepped for multiple isotopologue measurements on the IRMS Panorama at the University of Maryland Panorama Laboratory as follows. Prior to purification, methane concentration in head space gas was measured using a Shimadzu GC8A with FID. Methane from samples with high nitrogen concentrations was preconcentrated by freezing the sample into a liquid nitrogen-cooled U-trap packed with 100-120 mesh particle size HayeSep D porous polymer and then warming the trap to –115°C using an ethanol slush to release most N_2. The preconcentrated methane plus nitrogen was then transferred to the injection loop filled with silica gel and liquid nitrogen-cooled of a SRI 310C gas chromatograph, from which it was injected into a 1/8" diameter 25 Ft mol sieve 5 Å column at 54°C in a He carrier. The methane peak was detected using a TCD and then trapped in a liquid nitrogen-cooled collection loop filled with silica gel. The collector loop was isolated and residual helium was pumped away while the loop was kept at liquid nitrogen temperature, which retained

only the purified methane. The methane yield was determined monometrically, and the methane was transferred to a liquid-nitrogen cooled sample tube containing silica gel. After purification, methane samples were analyzed using the Panorama high resolution mass spectrometer (Nu Instrument) at The University of Maryland – College Park.

Methane was measured using sample standard bracketing relative to a working gas (UMD-1) and ratio data are reported in per mil using the δ and Δ notations, respectively:

$$\delta^{i} = \left(\frac{{}^{i}R_{sample}}{{}^{i}R_{standard}} - 1\right) \quad (13)$$

and

$$\Delta_{j} = \left(\frac{{}^{j}R_{sample}}{{}^{j}R_{stochastic}} - 1\right) \quad (14)$$

where ${}^{i}R$ or ^{j}R represent the isotope ratio by number *i*, including $^{13}C/^{12}C$ and D/H, or isotopologue ratio *j*, including $^{13}CH_3D/^{12}CH_4$ or $^{12}CH_2D_2/^{12}CH_4$, standards are Standard Mean Ocean Water (SMOW) for D/H and Pee Dee Belemnite (PDB) for $^{13}C/^{12}C$, and stochastic refers to the stochastic isotopologue ratio for the sample.

DISCUSSION

To partition and compare the percent microbial methane (PMM) throughout the sediment columns, we constructed isotopic mixing models that take into consideration $\delta^{13}C$, δD, $\Delta^{13}CH_3D$ and $\Delta\,^{12}CH_2D_2$ (17,18). The traditional isotope and hydrocarbon values of the methane sample from ~20m below the SMTZ of U1545 is within the well-constrained microbial fields (see Figure 4a-b) (19–22). Thus, for the microbial endmember, we chose $\delta^{13}C$ and δD values that are slightly more negative ($\delta^{13}C$ = -80 vs. -76‰; δD =-225 vs -200‰). Energy-rich environments where methane is rapidly produced, such as axenic microbial cultures and natural samples from

boreal lake wetlands, yield disequilibrated methane isotopologue compositions ($\Delta^{13}CH_3D$ = -4 to 4, $\Delta^{12}CH_2D_2$ = -60 to -15), credited to kinetic effects (23–26). Methane isotopologues can only be used as geothermometers at equilibrium, thus temperatures cannot be assigned at disequilibrated values. However, in energy-starved environments, such as the marine subsurface, methanogenesis and anaerobic oxidation of methane (AOM) may produce or cycle methane towards near equilibrium isotopologue values. This is suggested to be a result of high enzymatic reversibility caused by limited substrate and energy availability (27–32). Notice that while methane samples that fall within the 'microbial' fields of U1545 and U1547 have the expected near-equilibrium isotopologue values of the deep subsurface, those from U1546 fall within the region associated with energy-rich environments. As such, we chose microbial endmember isotopologues with disequilibrated values for U1546 ($\Delta^{13}CH_3D$ = 1, $\Delta^{12}CH_2D_2$ = -30), and near-equilibrium values for U1545 ($\Delta^{13}CH_3D$ = 5.3, $\Delta^{12}CH_2D_2$ = 5) and U1547 ($\Delta^{13}CH_3D$ = 4.3, $\Delta^{12}CH_2D_2$ = 5). The different $\Delta^{13}CH_3D$ values for U1545 and U1547 reflect their different temperature gradients.

Non-microbial methane can be of thermogenic or abiotic origin. Hence, we created two mixing models: with microbial and thermogenic endmembers; with microbial and abiotic endmembers (see Supplemental Figure 1-3). Thermogenic methane can yield either equilibrium or disequilibrium signatures, depending on whether it is sourced from mature or immature, respectively, hydrocarbon precurors (26,33). We know pyrolysis products in Guaymas sediments are thermally mature, and laboratory studies have assigned $\delta^{13}C$ values of -20 to -23‰ (8,34,35). As such, we use the following values for the thermogenic endmember: $\delta^{13}C$ = 20‰, δD = -150‰, $\Delta^{13}CH_3D$ = 1.6, $\Delta^{12}CH_2D_2$ = -2. Geothermal abiotic processes yield light $\delta^{13}C$ (-30 to -10‰) and δD (-200 to -50‰) signatures (36) and isotopologue signatures at or near high

temperature equilibrium ($\Delta^{13}CH_3D$ = 0 to 2) (27,37). Thus, for our abiotic endmember we assign $\delta^{13}C$ = 20‰, δD = -100‰, $\Delta^{13}CH_3D$ = 0.5, $\Delta^{12}CH_2D_2$ = -3. By taking the average of the two mixing models for each site (Supplemental Figure 1-3) and extrapolating the results up to the SMTZ and down the sediment column, we compare percent microbial methane (PMM) downcore and across sites (Figure 5).

References

1. de la Lanza-Espino G, Soto L. Sedimentary geochemistry of hydrothermal vents in Guaymas Basin, Gulf of California, Mexico - ScienceDirect [Internet]. 1999 [cited 2022 May 23]. Available from: https://www.sciencedirect.com/science/article/pii/S088329279800064X

2. Lizarralde D, Axen GJ, Brown HE, Fletcher JM, González-Fernández A, Harding AJ, et al. Variation in styles of rifting in the Gulf of California. Nature. 2007 Jul;448(7152):466–9.

3. Einsele G, Gieskes JM, Curray J, Moore DM, Aguayo E, Aubry MP, et al. Intrusion of basaltic sills into highly porous sediments, and resulting hydrothermal activity. Nature. 1980 Jan;283(5746):441–5.

4. Martens C. Generation of short chain acid anions in hydrothermally altered sediments of the Guaymas Basin, Gulf of California - ScienceDirect [Internet]. 1990 [cited 2022 May 23]. Available from: https://www.sciencedirect.com/science/article/pii/0883292790900376

5. Von Damm KL, Edmond JM, Grant B, Measures CI, Walden B, Weiss RF. Chemistry of submarine hydrothermal solutions at 21 °N, East Pacific Rise. Geochim Cosmochim Acta. 1985 Nov 1;49(11):2197–220.

6. Whelan JK, Hunt JM. C1-C8 hydrocarbons in DSDP Leg 64 Holes sediments [Internet]. Supplement to: Whelan, JK; Hunt, JM (1982): C1-C8 hydrocarbons in Leg 64 sediments, Gulf of California. In: Curray, JR; Moore, DG; et al. (eds.), Initial Reports of the Deep Sea Drilling Project (U.S. Govt. Printing Office), 64, 763-779, https://doi.org/10.2973/dsdp.proc.64.123.1982. PANGAEA; 1982. Available from: https://doi.pangaea.de/10.1594/PANGAEA.818767

7. Bradley JA, Arndt S, Amend JP, Burwicz E, Dale AW, Egger M, et al. Widespread energy limitation to life in global subseafloor sediments. Sci Adv. 2020;6(32):eaba0697.

8. Pearson A, Seewald JS, Eglinton TI. Bacterial incorporation of relict carbon in the hydrothermal environment of Guaymas Basin. Geochim Cosmochim Acta. 2005 Dec;69(23):5477–86.

9. Teske A, Callaghan AV, LaRowe DE. Biosphere frontiers of subsurface life in the sedimented hydrothermal system of Guaymas Basin. Front Microbiol [Internet]. 2014 [cited 2022 Apr 17];5. Available from: https://www.frontiersin.org/article/10.3389/fmicb.2014.00362

10. Teske A, Lizarralde D, Höfig T. Guaymas Basin Tectonics and Biosphere [Internet]. Expedition 385 Scientists, editor. International Ocean Discovery Program; 2021 [cited 2022 Apr 6]. (Proceedings of the International Ocean Discovery Program; vol. 385). Available from: http://publications.iodp.org/proceedings/385/385title.html

11. Lizarralde D, Soule SA, Seewald JS, Proskurowski G. Carbon release by off-axis magmatism in a young sedimented spreading centre. Nat Geosci. 2011 Jan;4(1):50–4.

12. Teske A, Lizarralde D, Höfig T. Site U1545 [Internet]. International Ocean Discovery Program; 2021 [cited 2022 Nov 28]. (Proceedings of the International Ocean Discovery Program). Available from: http://publications.iodp.org/proceedings/385/103/385_103.html

13. Teske A, Lizarralde D, Höfig T. Site U1546 [Internet]. International Ocean Discovery Program; 2021 [cited 2022 Dec 2]. (Proceedings of the International Ocean Discovery Program). Available from: http://publications.iodp.org/proceedings/385/104/385_104.html

14. Teske A, McKay LJ, Ravelo AC, Aiello I, Mortera C, Núñez-Useche F, et al. Characteristics and Evolution of sill-driven off-axis hydrothermalism in Guaymas Basin – the Ringvent site. Sci Rep. 2019 Sep 25;9(1):13847.

15. Teske A, Lizarralde D, Höfig T. Sites U1547 and U1548 [Internet]. International Ocean Discovery Program; 2021 [cited 2022 Dec 2]. (Proceedings of the International Ocean Discovery Program). Available from: http://publications.iodp.org/proceedings/385/105/385_105.html

16. D'Hondt S, Jørgensen BB, Miller DJ, Batzke A, Blake R, Cragg BA, et al. Distributions of microbial activities in deep subseafloor sediments. Science. 2004;306(5705):2216–21.

17. Giunta T, Young ED, Warr O, Kohl I, Ash JL, Martini A, et al. Methane sources and sinks in continental sedimentary systems: New insights from paired clumped isotopologues 13CH3D and 12CH2D2. Geochim Cosmochim Acta. 2019 Jan;245:327–51.

18. Zhang N, Snyder GT, Lin M, Nakagawa M, Gilbert A, Yoshida N, et al. Doubly substituted isotopologues of methane hydrate (13CH3D and 12CH2D2): Implications for methane clumped isotope effects, source apportionments and global hydrate reservoirs. Geochim Cosmochim Acta. 2021 Dec 15;315:127–51.

19. Etiope G, Sherwood Lollar B. Abiotic methane on Earth. Rev Geophys. 2013;51(2):276–99.

20. Milkov AV, Etiope G. Revised genetic diagrams for natural gases based on a global dataset of >20,000 samples. Org Geochem. 2018 Nov 1;125:109–20.

21. Whiticar MJ. Carbon and hydrogen isotope systematics of bacterial formation and oxidation of methane. Chem Geol. 1999 Sep 30;161(1):291–314.

22. Bernard B, Brooks J, Sackett W. Light hydrocarbons in recent Texas continental shelf and slope sediments. J Geophys Res. 1978 Jan 1;83:4053–61.

23. Gruen DS, Wang DT, Könneke M, Topçuoğlu BD, Stewart LC, Goldhammer T, et al. Experimental investigation on the controls of clumped isotopologue and hydrogen isotope ratios in microbial methane. Geochim Cosmochim Acta. 2018 Sep 15;237:339–56.

24. Taenzer L, Labidi J, Masterson AL, Feng X, Rumble D, Young ED, et al. Low Δ 12 CH 2 D 2 values in microbialgenic methane result from combinatorial isotope effects. Geochim Cosmochim Acta. 2020 Sep;285:225–36.

25. Wang DT, Gruen DS, Lollar BS, Hinrichs KU, Stewart LC, Holden JF, et al. Methane cycling. Nonequilibrium clumped isotope signals in microbial methane. Science. 2015 Apr 24;348(6233):428–31.

26. Young ED, Kohl IE, Lollar BS, Etiope G, Rumble D, Li (李姝宁) S, et al. The relative abundances of resolved l2CH2D2 and 13CH3D and mechanisms controlling isotopic bond ordering in abiotic and biotic methane gases. Geochim Cosmochim Acta. 2017 Apr 15;203:235–64.

27. Ash JL, Egger M, Treude T, Kohl I, Cragg B, Parkes RJ, et al. Exchange catalysis during anaerobic methanotrophy revealed by 12CH2D2 and 13CH3D in methane. Geochem Perspect Lett. 2019 Apr;26–30.

28. Douglas PM, Gonzalez Moguel R, Walter Anthony KM, Wik M, Crill PM, Dawson KS, et al. Clumped isotopes link older carbon substrates with slower rates of methanogenesis in northern lakes. Geophys Res Lett. 2020;47(6):e2019GL086756.

29. Gropp J, Iron MA, Halevy I. Theoretical estimates of equilibrium carbon and hydrogen isotope effects in microbial methane production and anaerobic oxidation of methane. 2022;69.

30. Lalk E, Pape T, Gruen DS, Kaul N, Karolewski JS, Bohrmann G, et al. Clumped methane isotopologue-based temperature estimates for sources of methane in marine gas hydrates and associated vent gases. Geochim Cosmochim Acta. 2022 Jun 15;327:276–97.

31. Ono S, Rhim JH, Gruen DS, Taubner H, Kölling M, Wegener G. Clumped Isotopologue Fractionation by Microbial Cultures Performing the Anaerobic Oxidation of Methane. 2020 Sep 1 [cited 2022 Jun 28]; Available from: https://chemrxiv.org/engage/chemrxiv/article-details/60c74f9ebb8c1a7e763db96a

32. Ono S, Rhim JH, Ryberg EC. Rate limits and isotopologue fractionations for microbial methanogenesis examined with combined pathway protein cost and isotopologue flow network models. Geochim Cosmochim Acta. 2022 May 15;325:296–315.

33. Dong G, Xie H, Formolo M, Lawson M, Sessions A, Eiler J. Clumped isotope effects of thermogenic methane formation: Insights from pyrolysis of hydrocarbons. Geochim Cosmochim Acta. 2021 Jun;303:159–83.

34. Simoneit BRT. Hydrothermal petroleum: genesis, migration, and deposition in Guaymas Basin, Gulf of California. Can J Earth Sci. 1985;22(12):1919–29.

35. Seewald JS, Seyfried WE, Shanks WC. Variations in the chemical and stable isotope composition of carbon and sulfur species during organic-rich sediment alteration: An experimental and theoretical study of hydrothermal activity at guaymas basin, gulf of california. Geochim Cosmochim Acta. 1994 Nov 1;58(22):5065–82.

36. Shuai Y, Etiope G, Zhang S, Douglas PMJ, Huang L, Eiler JM. Methane clumped isotopes in the Songliao Basin (China): New insights into abiotic vs. biotic hydrocarbon formation. Earth Planet Sci Lett. 2018 Jan 15;482:213–21.

37. Young ED. A Two-Dimensional Perspective on CH4 Isotope Clumping: Distinguishing Process from Source. In: Orcutt BN, Daniel I, Dasgupta R, editors. Deep Carbon [Internet]. 1st ed. Cambridge University Press; 2019 [cited 2022 May 17]. p. 388–414. Available from: https://www.cambridge.org/core/product/identifier/9781108677950%23CN-bp-13/type/book_part

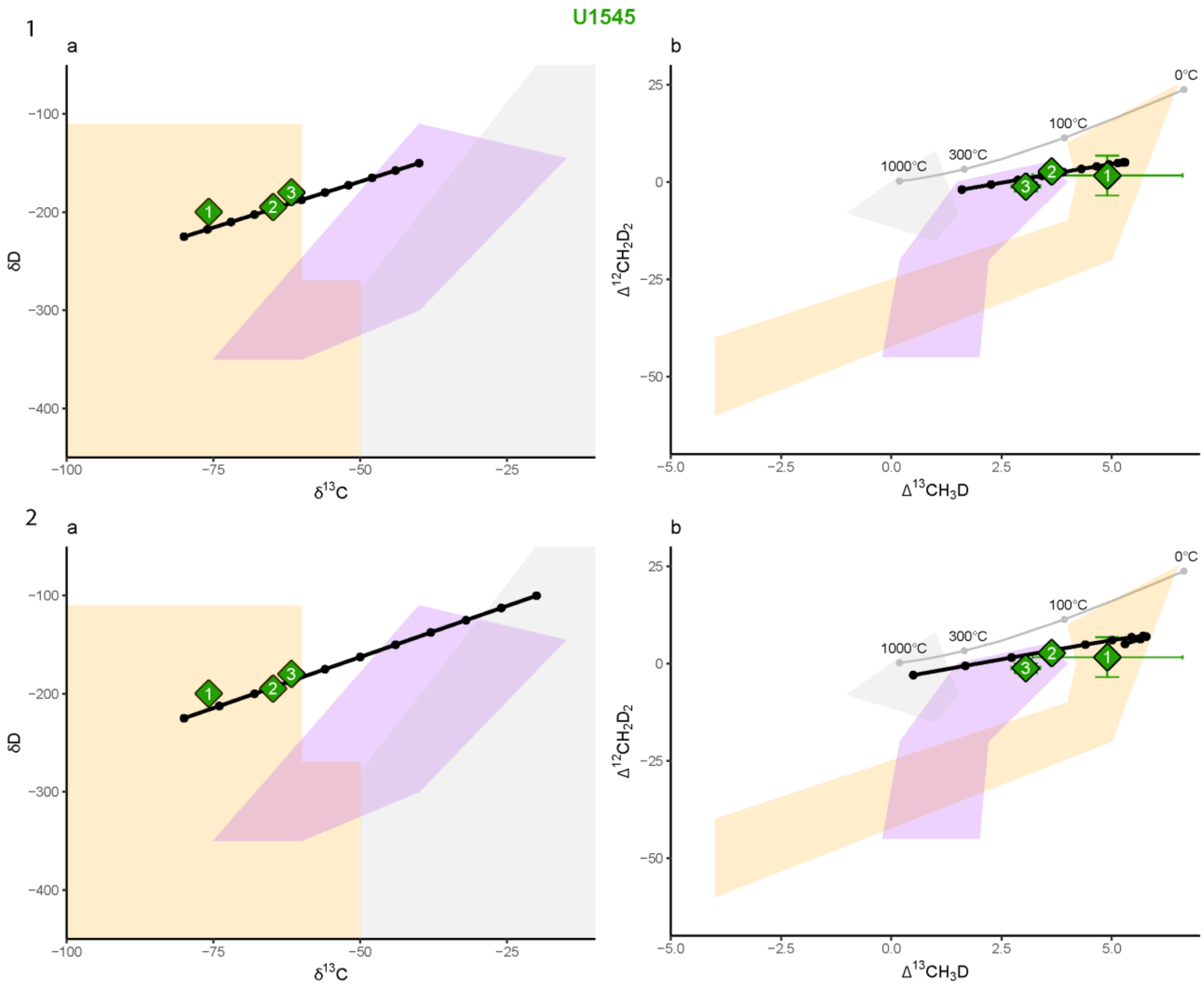

Supplementary Figure 1. **Mixing models (thick black line) to fit methane isotope measurements from site U1545** with (1) a microbial and a thermogenic endmember (2) a microbial and abiotic endmember; using (a) δ13C and δD values and (b) δ13C vs. δD values in combination with Δ13CH3D vs Δ 12CH2D2 values. Colored fields represent methane providence: orange for microbial, purple for thermogenic, grey for abiotic.

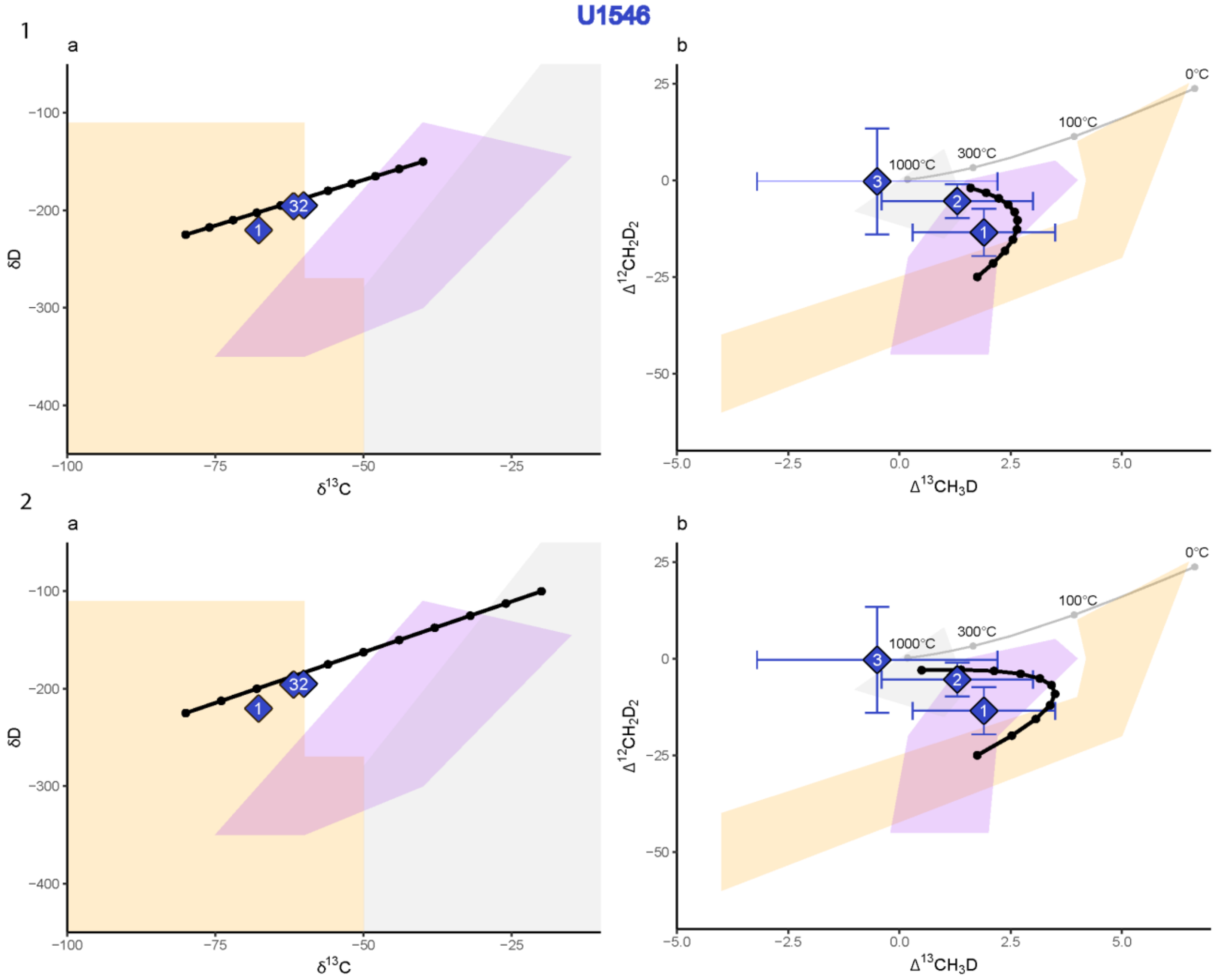

Supplementary Figure 2. **Mixing models (thick black line) to fit methane isotope measurements from site U1546** with (1) a microbial and a thermogenic endmember (2) a microbial and abiotic endmember; using (a) δ13C and δD values and (b) δ13C vs. δD values in combination with Δ13CH3D vs Δ 12CH2D2 values. Colored fields represent methane providence: orange for microbial, purple for thermogenic, grey for abiotic.

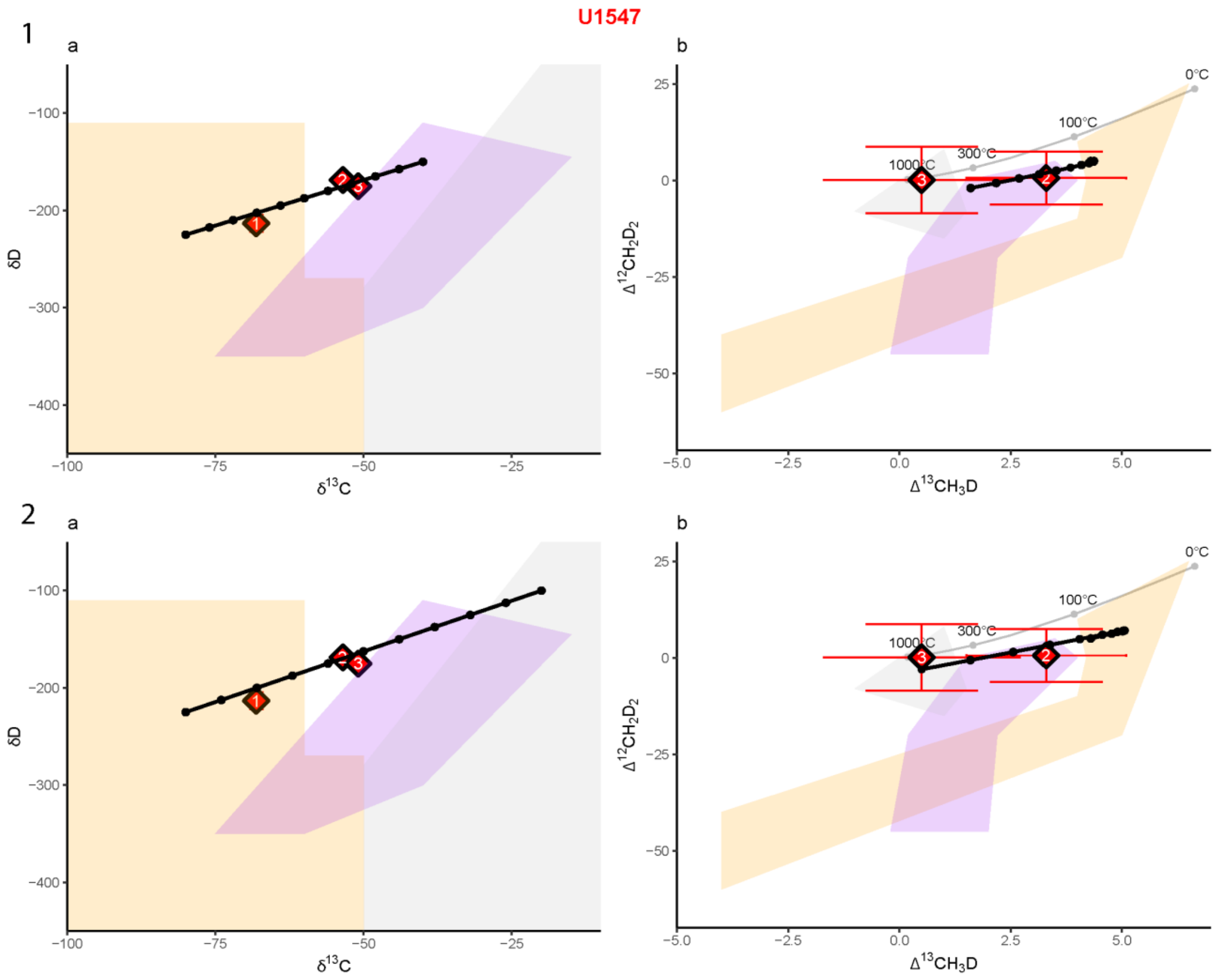

Supplementary Figure 3. **Mixing models (thick black line) to fit methane isotope measurements from site U1547** with (1) a microbial and a thermogenic endmember (2) a microbial and abiotic endmember; using (a) δ13C and δD values and (b) δ13C vs. δD values in combination with Δ13CH3D vs Δ 12CH2D2 values. Colored fields represent methane providence: orange for microbial, purple for thermogenic, grey for abiotic.

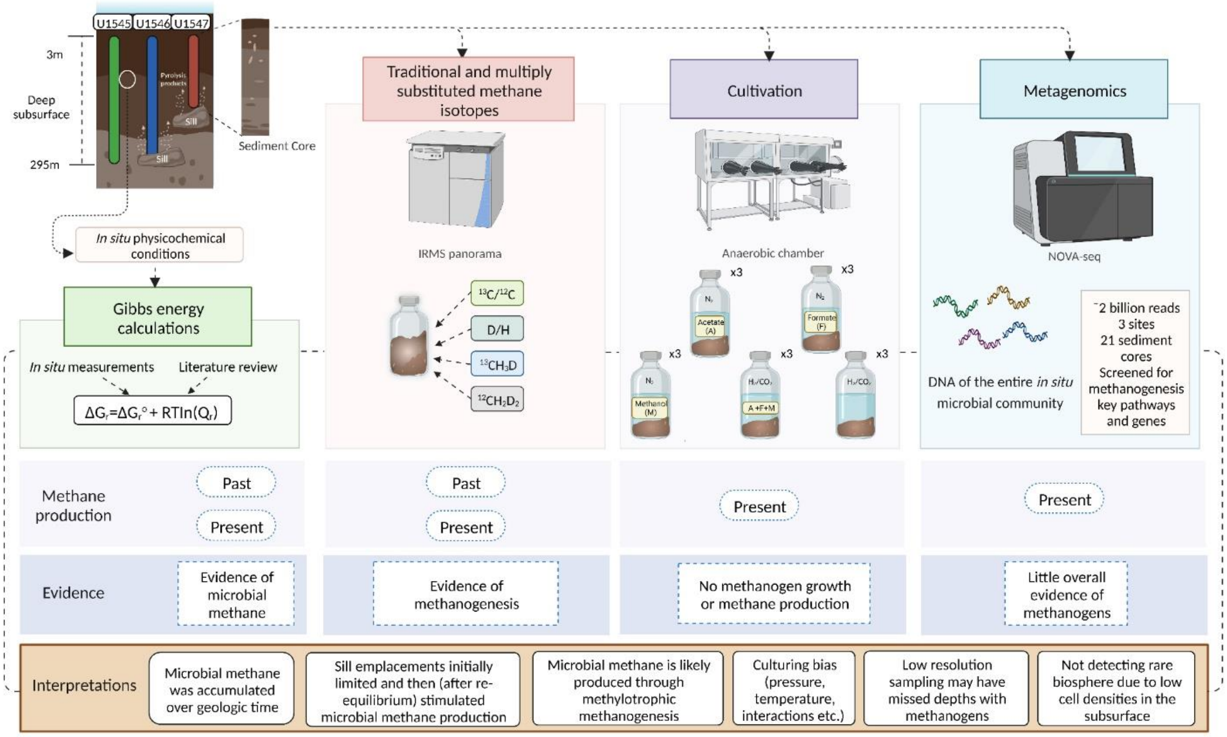

Supplementary Figure 4: **Graphical representation of methods, outcomes, and interpretations from this study.** This figure is a continuum of the graphical abstract. The top row of panels represents the techniques utilized, the second row of panels tells whether the technique can speak to methane production in geologic past or in the modern, the third row is whether the technique yielded evidence for methanogens or microbial methane. The final row is interpretations of the results or "evidence" found in this study.
